# The Incidence of Bacteremia and Risk Factors of Post-Radiofrequency Ablation Fever for Patients with Hepato-Cellular Carcinoma

**DOI:** 10.3390/cancers13215303

**Published:** 2021-10-22

**Authors:** Po-Yueh Chen, Tsung-Jung Tsai, Hsin-Yi Yang, Chu-Kuang Chou, Li-Jen Chang, Tsung-Hsien Chen, Ming-Tse Hsu, Chien-Chung Fang, Chang-Chao Su, Yu-Ling Lin, Yu-Min Feng, Chi-Yi Chen

**Affiliations:** 1 Department of Internal Medicine, Ditmanson Medical Foundation Chia-Yi Christian Hospital, Chia-Yi 60002, Taiwan; hdilwy7@gmail.com (P.-Y.C.); 02601@cych.org.tw (T.-J.T.); vacinu@gmail.com (C.-K.C.); cych07235@gmail.com (L.-J.C.); cych13794@gmail.com (T.-H.C.); 01686@cych.org.tw (M.-T.H.); 01705@cych.org.tw (C.-C.F.); 06155@cych.org.tw (C.-C.S.); 02557@cych.org.tw (Y.-L.L.); fengyumin2@gmail.com (Y.-M.F.); 2 Clinical Medicine Research Center, Ditmanson Medical Foundation Chia-Yi Christian Hospital, Chia-Yi 60002, Taiwan; cych13018@gmail.com; 3 Min-Hwei Junior College of Health Care Management, Tainan 73658, Taiwan

**Keywords:** radiofrequency ablation, hepatocellular carcinoma, fever

## Abstract

**Simple Summary:**

Post-radiofrequency ablation (Post-RFA) fever is a common minor complication of radiofrequency ablation in patients with hepatocellular carcinoma. We aim to evaluate the risk factors and positive blood culture rate of patients with post-RFA fever. We demonstrated that when younger people with a low serum albumin level underwent general anesthesia, the tumor numbers and tumor sizes were associated with a higher rate of post-RFA fever. A low incidence of bacteremia (4.8%) among patients with post-RFA fever was a novel finding which might indicate that prophylactic antibiotics are unnecessary for patients with hepatocellular carcinoma before ablation.

**Abstract:**

Post-radiofrequency ablation (RFA) fever is a self-limited complication of RFA. The correlation between post-RFA fever and bacteremia and the risk factors associated with post-RFA fever have not been evaluated. Patients with newly diagnosed or recurrent hepatocellular carcinoma who underwent ultrasonography-guided RFA between April 2014 and February 2019 were retrospectively enrolled. Post-RFA fever was defined as any episode of body temperature >38.0 °C after RFA during hospitalization. A total of 272 patients were enrolled, and there were 452 applications of RFA. The frequency of post-RFA fever was 18.4% (83/452), and 65.1% (54/83) of post-RFA fevers occurred on the first day after ablation. Patients with post-RFA fever had a longer hospital stay than those without (9.06 days vs. 5.50 days, *p* < 0.001). Only four (4.8%) patients with post-RFA fever had bacteremia. The independent factors associated with post-RFA fever were younger age (adjusted odds ratio (OR) = 0.96, 95% CI, 0.94–0.99, *p* = 0.019), low serum albumin level (adjusted OR = 0.49, 95% CI, 0.25–0.95, *p* = 0.036), general anesthesia (adjusted OR = 2.06, 95% CI, 1.15–3.69, *p* = 0.015), tumor size (adjusted OR = 1.52, 95% CI, 1.04–2.02, *p* = 0.032), and tumor number (adjusted OR = 1.71, 95% CI, 1.20–2.45, *p* = 0.003).

## 1. Introduction

Hepatocellular carcinoma (HCC) is the most common type of primary liver cancer and the fourth leading cause of cancer-related deaths worldwide [1]. HCC is associated with cirrhosis, chronic viral hepatitis, alcohol consumption, and nonalcoholic steatohepatitis [2].

Radiofrequency ablation (RFA) is one of the curative treatments for patients with HCC. RFA provides local tumor control, disease-free survival, and overall survival comparable to hepatic resection for patients with very early-stage or early-stage HCC. Moreover, RFA is associated with fewer complications than surgical resection for HCC in randomized controlled trials and meta-analyses [3,4,5]. In order to generate a larger ablation zone, multiple-electrode switching RFA has been performed for medium- or large-sized HCC in recent years [6,7]. Real-time virtual sonography and the creation of artificial ascites or pleural effusion have also been used to increase the conspicuity of tumors [8,9,10]. However, the RFA electrode tip can generate an electrical current that produces local heat, with temperatures reaching 60–100 °C, which leads to tissue necrosis [11]. Post-RFA complications include fever, wound pain, nausea, and vomiting; they are known as post-RFA syndrome and occur in 35% of patients [12,13,14]. The etiology of the post-RFA syndrome is uncertain and controversial, but a systemic reaction after thermal ablation might be the major cause [14]. Fever after ablation is common and the most concerning for patients and physicians.

Prolonged post-RFA fever is also associated with the early recurrence of HCC in patients who undergo ablation [15]. Although most post-RFA fever is self-limited, it raises concerns about performing blood culture testing, prescribing empirical antibiotics, and even prolonging hospitalization. There is little evidence on whether different techniques in RFA, such as creating artificial ascites or using overlapping or multiple-electrode switching RFA, would induce a higher rate of post-RFA fever. Moreover, the rate of positive blood culture test results, which is necessary to identify a true infection, has never been evaluated among patients with post-RFA fever. The risk factors associated with post-RFA fever have also not been identified. Therefore, we aimed to evaluate the incidence of post-RFA fever in the era of advanced RFA techniques. We also examined the incidence of positive blood culture test results and analyzed the risk factors of post-RFA fever in HCC patients who underwent radiofrequency ablation.

## 2. Materials and Methods

### 2.1. Patients

Patients with newly diagnosed or recurrent HCC who underwent ultrasonography-guided percutaneous monopolar RFA between April 2014 and February 2019 at the Ditmanson Medical Foundation Chia-Yi Christian Hospital in Taiwan were enrolled retrospectively. Patients who received combined RFA and transarterial chemoembolization (TACE), RFA with percutaneous ethanol injection, or intraoperative RFA were excluded. The diagnosis of HCC was based on typical imaging findings according to the American Association for the Study of Liver Diseases Practice Guidelines or the pathological report. Patients who were pathologically diagnosed with combined HCC with cholangiocarcinoma, sarcomatoid HCC, or other poorly differentiated carcinomas were excluded. The use of prophylactic antibiotics routinely in patients with HCC before ablation was weakly recommended (class IIb) because there was a low level of evidence (grade C) in the clinical practice guidelines [16]. Therefore, prophylactic antibiotics were only prescribed for patients with a biliary surgical history in our hospital. In order to evaluate the incidence of post-RFA fever, we also excluded patients who received prophylactic antibiotics before RFA or those with a fever before ablation. This study was approved by the Chia-Yi Christian Hospital Ethics Committee and conformed to the principles of the Declaration of Helsinki and the International Conference on Harmonization for Good Clinical Practice (IRB number IRB2021095, Chia-Yi Christian Hospital IRB). This retrospective observational study used deidentified and routine diagnosis/treatment data, thus CYCH-IRB- 2021095 approved the waiver of informed consent from all patients for being included in the study.

### 2.2. Study Design

We collected baseline characteristics and serum laboratory data, including platelet count, prothrombin time, albumin level, and total bilirubin level of patients each time they received RFA. We also collected data on tumor characteristics, such as the diameter of the largest ablated tumor and the number of ablated tumors. Additionally, information on the RFA procedure, including the procedure time, creation of artificial ascites, overlapping ablation, and use of multiple-electrode monopolar switching RFA, was also collected.

Multiple-electrode switching RFA was defined as the insertion of two or three monopolar electrodes in the targeted lesion and the generation of an electrical current by an automatic sequence. We defined overlapping ablation as more than one sequential electrode puncture into the targeted lesion during ablation. Artificial ascites were composed of 5% dextrose in 0.9% normal saline (D5-saline solution) and infused into the peritoneum using an ENDOPATH^®^ Pneumoneedle Insufflation Needle (Johnson&Johnson, NJ, USA). The patterns of anesthesia were also recorded and divided into local anesthesia, which was defined as a local subcutaneous lidocaine with an intravenous drip of fentanyl, and general anesthesia, which was administered by an anesthesiologist using intravenous propofol for induction, inhaled sevoflurane with a laryngeal mask for maintenance, and intermittent administration of muscle relaxants.

### 2.3. Post-RFA Assessment

**Figure 1:**
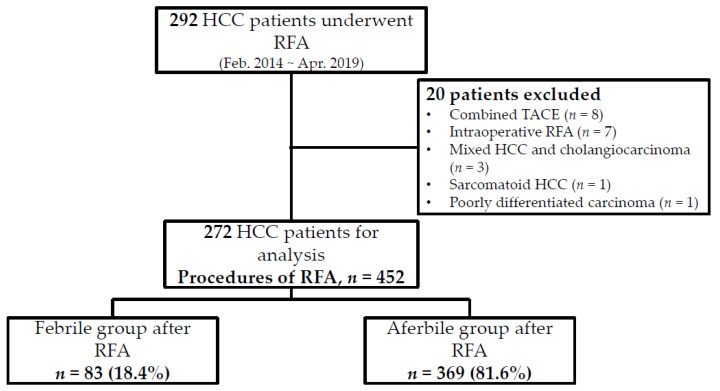
Algorithm of patient enrollment. Abbreviations: HCC, hepatocellular carcinoma; RFA, radiofrequency ablation.

Post-RFA fever was defined as any episode of body temperature higher than 38.0 °C after RFA during hospitalization. Patients with and without post-RFA fever were divided into febrile and afebrile groups, respectively (Figure 1). For patients with post-RFA fever, a blood culture test was performed when the patient developed a fever. The results of the blood culture test performed after the development of post-RFA fever were recorded, and we analyzed the characteristics of patients with positive blood culture test results. We also reviewed the electronic medical records and laboratory data of all enrolled patients within 7 days after discharge. Patients who had a positive blood culture test result during this period were recorded. We also assessed the length of stay for all enrolled patients at each admission.

### 2.4. Statistical Analysis

Baseline and tumor characteristics are presented as means and standard deviations or as percentages. The incidence of post-RFA fever is presented as a percentage and a 95% confidence interval (CI). Because a patient might receive multiple rounds of ablation, the baseline characteristics before ablation, the parameters of the RFA procedure, and the outcome after every ablation were noted each time as an isolated episode. Therefore, we used generalized estimating equations to analyze these longitudinal data with multiple observations. The Mann–Whitney U test and Pearson’s chi-squared analysis, or Fisher’s exact test were used to compare continuous and categorical variables, respectively. All assumed variables were included in the univariate and multivariate analyses. A two-tailed *p* < 0.05 was defined as statistical significance. All statistical analyses were performed using the Statistical Program for Social Sciences (SPSS Statistics Version 21.0, IBM Corp., Armonk, NY, USA).

## 3. Results

**Figure 2:**
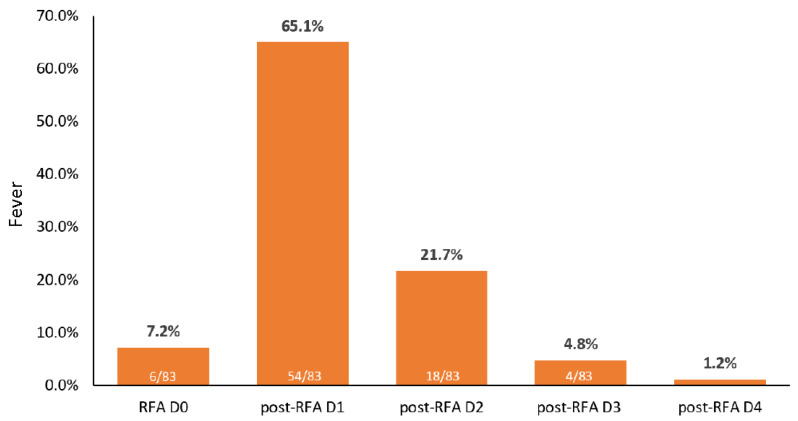
The frequency of post-radiofrequency ablation fever during hospitalization. Abbreviations: RFA, radiofrequency ablation; RFA D0, date of RFA; post-RFA D1, one day after RFA.

A total of 292 patients who received ultrasonography-guided monopolar RFA were screened retrospectively by their electronic medical record. Twenty patients were excluded due to combined TACE, intraoperative RFA, or mixed HCC and cholangiocarcinoma in the pathological report. Therefore, 272 patients who underwent RFA 452 times were included in the analysis. Among the 452 administrations of RFA, 83 resulted in post-RFA fever during admission (Figure 1). The frequency of post-RFA fever was 18.4% (83/452), and 65.1% (54/83) of post-RFA fevers occurred on the first day after ablation (Figure 2).

The demographic data, clinical characteristics, tumor characteristics, and detailed records of each procedure are shown in Table 1. The mean ages of the febrile and afebrile groups were 67.28 years and 68.90 years, respectively. Most patients had Child–Pugh stage A liver function (73.49% and 74.53% in the febrile and afebrile groups, respectively), and the mean diameter of the largest ablated tumor was less than 3.0 cm in both groups. Ablation was performed 210 times (46.5%, 210/452) with local anesthesia and 242 times (53.5%, 242/452) with general anesthesia. Of the 452 rounds of ablation, 162 used overlapping ablation (35.8%, 162/452), 70 used multiple-electrode switching RFA (15.5%, 70/452), and 119 used artificial ascites (26.3%, 119/452).

The univariate analyses of factors associated with post-RFA fever indicate that the male sex, serum albumin level, general anesthesia, tumor diameter, tumor number, and overlapping ablation were associated with post-RFA with statistical significance (*p* < 0.05) (Table 1). Additionally, the mean length of the hospital stay was longer in the febrile group than in the afebrile group, with statistical significance (9.06 days vs. 5.50 days, *p* < 0.001). In the multivariate analysis, the age (adjusted odds ratio (OR) = 0.96, 95% CI, 0.94–0.99, *p* = 0.019), serum albumin level (adjusted OR = 0.49, 95% CI, 0.25–0.95, *p* = 0.036), general anesthesia (adjusted OR = 2.06, 95% CI, 1.15–3.69, *p* = 0.015), tumor size (adjusted OR = 1.52, 95% CI, 1.04–2.02, *p* = 0.032), and tumor number (adjusted OR = 1.71, 95% CI, 1.20–2.45, *p* = 0.003) were independent factors associated with post-RFA fever at the time of ablation (Table 2).

Among the 83 cases in the febrile group, all patients received blood culture tests at the time of fever onset, and only four (4.8%, 4/83) had positive blood culture test results during hospitalization. After reviewing the medical records and laboratory data of all patients within 7 days after discharge, none had a positive blood culture test result, and only three patients visited the emergency room due to wound pain. Table 3 shows the characteristics of patients with a positive blood culture test result. There was no statistical significance of the association between tumor location and bacteremia in our analysis (Table 4). Three patients had Gram-negative bacilli bacteremia (two had Escherichia coli infections and one had a Klebsiella pneumoniae infection), and one patient was infected by Staphylococcus epidermidis. Of the four patients with bacteremia, one patient developed a liver abscess and received percutaneous drainage (culture: Klebsiella pneumoniae).

## 4. Discussion

This is a comprehensive study that focused on the factors associated with fever after RFA in HCC patients who did not receive prophylactic antibiotics. The frequency of post-RFA fever in our study was 18.4% (83/452). The incidence of post-RFA fever varies widely in different reports. In an earlier study by Carrafiello et al. [12], post-ablation syndrome, including fever, pain, or malaise, was a common minor complication that occurred in one third of patients who underwent ablation of abdominal tumors, and the rate of post-RFA fever (body temperature 37.5–38.5 °C) was approximately 8.5% (7/71) [12]. Recently, Park et al. [17] evaluated the early complications of 1211 patients with HCC who underwent RFA, of whom 4.1% had post-RFA syndrome, including fever and pain [17]. In a meta-analysis by Luo et al. [18], the incidence of post-RFA fever ranged from 11.7% to 70.5%, with an average of 37.3% [18,19,20,21]. Another study by Lin et al. [7] showed that 30.6% of patients with HCC who underwent multiple-electrode RFA developed post-RFA fever [7]. Nevertheless, these studies did not report on the administration of prophylactic antibiotics or have a clear definition of post-RFA fever. In a study by Tateishi et al. [22], antibiotics were administered before and after RFA, and they continued antibiotics for patients with a body temperature > 37.5 °C [22]. However, the incidence of post-RFA fever was not mentioned in Tateishi’s study.

Although post-RFA fever is considered to be self-limited and a minor complication of ablation, if a patient has post-RFA fever during hospitalization, physicians must determine whether to administer prophylactic or empirical antibiotics, perform blood culture tests, or even postpone the patient’s discharge. Our study is the first to reveal the incidence of post-RFA in HCC patients who did not receive prophylactic antibiotics. Moreover, all febrile patients in our study received a blood culture test, and only 4.8% of patients had a positive blood culture test result. In an earlier study, Bhatia et al. [23] revealed that the incidence of liver abscess after RFA in patients with HCC was 0.8% per session, and none of the enrolled patients received prophylactic antibiotics [23]. Therefore, they concluded that the administration of prophylactic antibiotics might be unnecessary for patients undergoing RFA due to the rare occurrence of liver abscess. However, liver abscess is considered a major complication of RFA, and we might underestimate the proportion of true infection that should be treated with antibiotics for patients undergoing ablation. The low incidence of bacteremia (4.8%) in HCC patients with post-RFA fever in our study might indicate that the overall occurrence of bacteremia was only 0.88% (4/452) per session of ablation. This result strongly suggests that the administration of prophylactic antibiotics is unnecessary, except for those patients with a high risk of infection, such as those with a pre-existing biliary abnormality.

In our study, younger age, low serum albumin level, general anesthesia, larger tumors, and higher tumor number were the independent factors associated with post-RFA fever in the multivariate analysis. In addition to coagulation necrosis of the central part of the tumor by RFA, inflammatory infiltration occurred in the transitional zone adjacent to the central area. Elevated proinflammatory cytokines, including interleukin (IL)-1β, IL-6, IL-8, and tumor necrosis factor-α, were found not only in the adjacent ablated tissue but also in the peripheral blood after RFA [24,25,26]. Therefore, fever after ablation could be triggered by these cytokines. The ablation of larger tumors and more tumors would induce the accumulation of more cytokines, which might increase the incidence of post-RFA fever. Compared to older patients, younger patients can have a stronger reaction to the inflammatory process after ablation and thus have a higher rate of post-RFA fever. The type of anesthesia for radiofrequency ablation varies widely in different institutions, ranging from local anesthesia to general anesthesia [27]. Administering the general anesthesia using a laryngeal mask airway (LMA) could reduce postoperative hoarseness and sore throat and shorten the stay in the post-anesthesia recovery unit [28]. However, administering it via an LMA also decreases the lower esophageal sphincter tone and is associated with a greater incidence of gastric insufflation, which might induce regurgitation or aspiration [29,30,31]. In our study, general anesthesia was administered through an LMA. The increased risk of aspiration might be associated with the higher incidence of post-RFA fever in patients who receive general anesthesia.

The strengths of our study were that we clearly defined post-RFA fever and that none of the enrolled patients received prophylactic antibiotics. The low incidence of bacteremia in HCC patients with post-RFA fever (4.8%) and the per-session incidence of bacteremia (0.88%) are novel findings that might prevent the unnecessary administration of prophylactic antibiotics to HCC patients undergoing RFA. We also analyzed the risk factors of post-RFA fever: younger age, higher tumor number, and general anesthesia were the independent factors for a higher risk of post-RFA fever. Nevertheless, there were some limitations in our study. First, we only recorded fever episodes during hospitalization; therefore, fevers occurring after discharge were missed, and the overall incidence of post-RFA fever might be underestimated. Second, despite reviewing the electronic medical record within 7 days after discharge for all patients, some patients with asymptomatic bacteremia who did not seek medical help would also be missed. Third, the patients enrolled in our study were all diagnosed with HCC and received RFA. This result might not be generalizable to other types of intrahepatic neoplasms or different devices for ablation of intrahepatic tumors.

## 5. Conclusions

In conclusion, post-RFA fever is a common minor complication in HCC patients undergoing RFA and can prolong the length of hospitalization. The incidence of bacteremia in HCC patients with post-RFA fever was low (4.8%); therefore, prophylactic antibiotics might be unnecessary, particularly because the assumed incidence of bacteremia was only 0.88% per session of RFA. The independent risk factors of post-RFA fever were younger patients, low serum albumin level, general anesthesia, larger tumors, and tumor number.

## Figures and Tables

**Table 1:** Univariate analysis of factors of post-RFA fever.

Characteristics	Febrile (*n* = 83)	Afebrile (*n* = 369)	*p-*Value
Age, year, mean (SD)	67.28 ± 9.23	68.90 ± 9.84	0.170
Male, *n* (%)	**37 (78.72)**	**128 (56.89)**	**0.005**
Total bilirubin, mg/dL, mean (SD)	0.84 ± 0.54	0.79 ± 0.59	0.375
Albumin, g/dL, mean (SD)	**3.7 ± 0.48**	**3.85 ± 0.52**	**0.020**
Platelet count, k/μL, mean (SD)	119.12 ± 56.58	119.64 ± 57.15	0.940
Child–Pugh stage			0.612
A (5–6)	61 (73.49)	275 (74.53)	
B (7–9)	13 (15.66)	43 (11.65)	
C (≥10)	0 (0.00)	3 (0.81)	
Anesthesia			**0.020**
Local	**29 (34.94)**	**181 (49.05)**	
General	**54 (65.06)**	**188 (50.95)**	
Procedure time, min	19.19 ± 10.49	17.11 ± 12.55	0.160
Tumor diameter, cm	**2.76 ± 0.86**	**2.51 ± 1.03**	**0.037**
Tumor number			**0.003**
1	**47 (56.63)**	**263 (71.27)**	
2	**24 (28.92)**	**83 (22.49)**	
3	**7 (8.43)**	**19 (5.15)**	
4	**3 (3.61)**	**4 (1.08)**	
5	**2 (2.41)**	**0 (0.00)**	
Overlapping ablation, *n* (%)	**39 (46.99)**	**123 (33.33)**	**0.019**
Multiple-electrode switching ablation, *n* (%)	15 (18.07)	55 (14.91)	0.471
Ascites			
Pre-existing, *n* (%)	18 (21.69)	54 (14.63)	0.113
Artificial, *n* (%)	26 (31.33)	93 (25.20)	0.253
Length of hospitalization, days	**9.06 ± 4.31**	**5.50 ± 3.21**	**<0.001**

Bold numbers indicate statistical significance (*p* < 0.05). Abbreviations: RFA, radiofrequency ablation; SD, standard deviation.

**Table 2:** Multivariate analysis of factors of post-RFA fever.

Characteristics	Adjusted OR (95% CI)	*p*-Value
Age	**0.96 (0.94–0.99)**	**0.019**
Male sex	1.51 (0.82–2.77)	0.187
Total bilirubin	0.81 (0.47–1.38)	0.427
Albumin	**0.49 (0.25–0.95)**	**0.036**
Platelet count	1.00 (1.00–1.01)	0.847
Child–Pugh stage	0.96 (0.82–1.12)	0.583
General anesthesia	**2.06 (1.15–3.69)**	**0.015**
Procedure time	0.98 (0.95–1.01)	0.118
Tumor diameter	**1.52 (1.04–2.22)**	**0.032**
Tumor number	**1.71 (1.20–2.45)**	**0.003**
Overlapping ablation	1.52 (0.86–2.67)	0.147
Multiple-electrode switching ablation	0.60 (0.26–1.36)	0.222
Pre-existing ascites	1.06 (0.50–2.24)	0.877
Artificial ascites	1.21 (0.71–2.08)	0.485

Bold numbers indicate statistical significance (*p* < 0.05). Abbreviations: CI, confidence interval; OR, odds ratio; RFA, radiofrequency ablation.

**Table 3:** Baseline characteristics of patients with positive blood culture.

Sex	Age	Tumor Character	Tumor Location	Fever Onset after RFA (Day)	Procedure Time (min)	Anesthesia	Pathogenic Bacteria	Abscess Formation	Hospitalization (Day)
M	74	Recurrence	S4	1	10	Local	*E. coli*	No	14
M	63	Fresh	S5,S8	1	18	Local	*E. coli*	No	12
M	71	Recurrence	S6	1	41	Local	*S. Epidermidis*	No	9
M	75	Fresh	S8	2	8	General	*K. Pneumoniae*	Yes	25

Abbreviation: *E. coli*, *Escherichia coli*; *K. Pneumoniae*, *Klebsiella pneumonia*; M, male; RFA, radiofrequency ablation; *S. Epidermidis*, *Staphylococcus epidermidis*.

**Table 4:** The association between tumor location and the occurrence of bacteremia in the febrile group.

Tumor Location	Crude OR (95% CI)	*p*-Value
S4	1.85 (0.17–19.55)	0.611
S5	0.93 (0.09–9.54)	0.957
S6	0.88 (0.09–8.42)	0.913
S8	2.92 (0.38–22.29)	0.301

Other sites of tumor location could not be analyzed due to limited event numbers. Abbreviation: CI, confidence interval; OR, odds ratio; S, segment.

## Data Availability

The data presented in this study are available on request from the corresponding author.
